# Transforming Beef By-products into Valuable Ingredients: Which Spell/Recipe to Use?

**DOI:** 10.3389/fnut.2016.00053

**Published:** 2016-11-30

**Authors:** Maeve Henchion, Mary McCarthy, Jim O’Callaghan

**Affiliations:** ^1^Teagasc, Food Research Centre Ashtown, Dublin, Ireland; ^2^Department of Food Business and Development, University College Cork, Cork, Ireland

**Keywords:** consumer, acceptance, rejection, food processing technologies, ideation, offal, by-products

## Abstract

Satisfying the increasing global demand for protein results in challenges from a supply perspective. Increased use of animal proteins, through greater use of meat by-products, could form part of the solution, subject to consumer acceptance. This research investigates consumer evaluations of food products that incorporate ingredients derived from offals that have been produced through a range of food processing technologies. Using focus groups incorporating product stimuli representing various combinations of offals, processing, and carrier products, the research finds that the physical state and perceived naturalness of the ingredients influences acceptance. It also highlights the impact of life experiences, linked to demographic characteristics, on interpretations and evaluations of products and processes. Ideational influences, i.e., knowledge of the nature or origin of the substance, are reasons for rejecting some concepts, with misalignment between nature of processing and the product resulting in rejection of others. Lack of perceived necessity also results in rejection. Alignment of ingredients with existing culinary practices and routines, communication of potential sensory, or other benefits as well as naturalness are factors likely to promote acceptance, and generate repeat purchase, in some consumer segments. Trust in oversight that the products are safe is a prerequisite for acceptance in all cases. These findings have implications for pathways to increase sustainability of beef production and consumption through increased use of beef by-products.

## Introduction

Global demand for food, particularly food of high protein content, is increasing. Satisfying this demand is a global challenge given the finite resources of the planet, the impact of increased production on the environment, and other current and future food sustainability challenges (1–3). Various strategies are proposed to address the supply side of this challenge. These fall into two generic categories: increasing output from existing resources but mitigating the negative impact of such production and utilizing alternative/novel sources of protein. Making better use of meat by-products straddles both strategies by potentially reducing the environmental impact of increased meat production as well as presenting new ingredients and products to consumers. Such a strategy could also respond to the need articulated by various authors (4, 5) to use animal proteins in a more responsible manner than is currently the case. Consumers do not however, accept such novel products without question and what may seem like a practical solution from a science as well as policy perspective may encounter resistance from consumers. Indeed there is a suggestion that consumers are not familiar with potential innovations in this area and as result may consider by-products as unhealthy and indeed waste (6). This study seeks to explore consumer attitudes toward food products that incorporate ingredients derived from beef by-products that have been produced through a range of food processing technologies.

The focus on beef by-products is warranted given that up to 56% of the total live weight of a beef animal can comprise non-meat components (7). Many of these components are fit for human consumption and contain high amounts of protein, essential amino acids, vitamins, minerals, antioxidants, and bioactive peptides (8, 9). However, for various reasons including regulatory issues, meat industry work practices, and cultural factors (5, 10, 11), a significant volume of such by-products result in low-value products and are often treated as waste with negative environmental and cost implications (12). Finding higher value end-uses for the volume of non-meat components that can arise from meat production will become increasingly important given a projected production increase of 200 million tons of meat by 2050 (13). Much research is ongoing in many countries to add value to meat by-products (14) including profiling the functional and compositional characteristics of by-products [e.g., Ref. (5)], developing processes to extract value added constituents from by-products [e.g., Ref. (10)], examining sensory aspects of products that contain by-products [e.g., Ref. (15)], and indeed into non-food uses including high value pharmaceutical, cosmetic and nutraceutical use (16), and lower value bioenergy uses (12).

## Factors Influencing Consumer Acceptance/Rejection of Foods

People tend to avoid unknown/unfamiliar products, for a range of reasons including the fear of ingesting toxins and other pathogens (17). This tendency, referred to as food neophobia, is not homogenous across foods, and can to be stronger in response to animal products than non-animal products, possibly as a result of the greater potential pathogenic threat posed by animal products (18). However, people are also sensation seekers and enjoy novelty, and need to consume a varied diet for adequate nutrition and health reasons (19). This phenomenon of the “omnivore’s dilemma” has led many researchers to ask the question: how will consumers react when faced with a product with which they are unfamiliar or which is different in some way: will they accept or reject it?

Two streams of research have emerged that consider consumer responses to unfamiliar foods and processes. One stream has considered consumer acceptance [e.g., Ref. (20)] while the second has examined consumer responses through the lens of rejection (21). Rozin and Fallon (21) drew attention to three important motives that lead to product rejection: negative sensory properties (distaste), harmful consequences (perceived danger), and “ideational.” Two of these, distaste and perceived danger, are particularly important to acceptance and indeed levels of demand (22). If danger is perceived, irrespective of any benefits, the product will be rejected. However, where safety is assured taste becomes critical. The third motive, “ideational” is concerned with knowledge of the nature or origin of the substance. The negative pole of this is disgust, whereby foods are rejected “because of what they are, where they came from, or their social history (e.g., who touched them or ate them)” [(22), p. 215]. Disgust encompasses textural and sensory properties that are disliked (e.g., tastes bad) as well as reminders of livingness/animalness, and a capacity to contaminate other food (23). Disgust not only relates to revulsion at the prospect of ingesting a product, it can also relate to the pre-ingestion phase including physical contact with the body during preparation, with consumers reluctant to touch or smell raw meat and meat cuts (24). As such disgusting items are offensive and objectionable. A second ideational reason for rejecting foods is inappropriateness (23). This relates to the idea of edibility is so far as rejection on this basis occurs for items that are not classified as foods within a given culture.

Where products are not rejected outright, consumer preferences for products are based on characteristics of a product. In the food domain in particular, humans have a “natural preference” and a strong desire for things that are natural. Natural preference can be based on ideational/moral beliefs in the superiority of nature as well as instrumental understanding. Rozin et al. (25) found causal links between perceived healthiness and a preference for natural foods, for example, however, they stress the importance of ideational factors when health and sensory properties are controlled for Rozin (26) and Evans et al. (27) highlight the influence of processing on perceived naturalness. Further they discuss the impact of the nature of the transformation (chemical transformations are concluded to produce more significant reductions in naturalness than physical transformations), the impact of mixing with other natural entities (small additions of an unlike natural entity causes a bigger reduction in naturalness than any mixture of like natural entities), and the principle of contagion on perceived naturalness. Unlike Rozin however, Evans et al. (27) highlight the impact of content, in terms of carrier product and ingredients, on naturalness.

Products can be presented to consumers in a range of physical forms; for solid raw materials, they can be presented in an original, solid, non-altered state, a semi-solid processed state, and as a processed liquid for example. Evidence suggests that the physical state of a product can influence bio-physical food consumption (e.g., intake and satiety) (28). However, more recent research also finds that the physical state of a product influences psychological factors, e.g., perceived healthiness, expected emotional experiences, expected satiety, and thus ultimately consumption decisions (28–31). The impact of the physical state of the product/ingredient on perceptions is illustrated in the so called “blender effect” whereby foods that are raw or in a less processed state are perceived as healthier and preferable to more highly processed foods, e.g., liquid form of a solid raw material is seen as less healthy than the solid raw material. However, it is not only the physical form that influences perceptions but also the processes used to create the product. Using mechanical processing, Szocs and Lefebvre (30) find that perceived healthiness is influenced not only by the physical state but also by the degree of processing suggested by the physical state. Gmuer et al. (29) who explore consumer responses to crickets presented as (1) flour, (2) deep-fried bits, (3) a snack mix comprising bits mixed with tortilla chips, and (4) deep-fried whole crickets, found that the degree of processing influenced willingness to eat insects. However, they also indicate that the relationship is more complex than suggested from previous research; whereas previous research suggested that a higher level of processing, with the insect ingredient being less visible, is associated higher levels of acceptance (32, 33), these researchers found a less “linear” relationship. Nonetheless research in this area clearly indicates that processing impacts on perceived naturalness and where an ingredient in unfamiliar or potentially unacceptable, processing can have the positive impact of making the ingredients more acceptable.

Research that examines the opportunity to substitute particular foodstuffs and change dietary habits identifies that familiarity and knowledge influence acceptance. Wansink (34) who examined American consumers’ inclusion of protein-rich offal into their diets during World War II highlights that food must not only taste nice and look, taste and feel as expected it must also be familiar. Similarly, Schölser et al. (31) in examining Dutch consumers’ meat substitution motivations and behavior identify product familiarity, cooking skills, and food knowledge as influencing factors. This leads us to consider the factors that might influence acceptance of beef offal as part of one’s diets in a high income market characterized by limited familiarity and experience of these offal.

## Methodology

A qualitative research approach was taken to provide insights into factors influencing consumers’ evaluations of novel or innovative food products derived from animal coproducts and ultimately the implications of this on acceptance. Qualitative research offers a means of understanding how attitudes form and evolve (35), and the multi-dimensional cognitive and emotive processes around evaluating novel food products. Given the newness of the concepts under investigation (from a consumer perspective) an approach that allowed discussion and exchange of ideas was central to observing what shaped attitudes, thus focus groups were used. As the quality of qualitative research is dependent on, among other factors, respondent characteristics, recruitment was based on clearly pre-defined exclusion and inclusion criteria presented in a screening questionnaire. Given the influence of age, gender, and social class on consumers’ risk and benefit perception of food, and thus on likely acceptance of products incorporating beef by-products, focus groups were selected to ensure diversity across these factors (Table 1). Moreover, recruitment of a diversity of consumers enhances the transferability and dependability of the research (36). Individual focus groups however comprised participants who were quite similar to facilitate participants sharing their views and attitudes and to encourage discussion (37, 38). Exclusion criteria relating to meat consumption and shopping behavior were included in the recruitment questionnaire. Furthermore, consumers were not recruited if they had partaken in a survey or focus group in the past 6 months in line with best practice and those directly involved in the food industry and marketing/advertising will be excluded due to their particular orientations and knowledge. All participants were screened and recruited by a professional field agency. To offset the time and costs involved in participating in the focus groups, a sum of €50 was given to each participant.

**Table 1:** **Composition of focus groups**.

Focus group no.	Participants	Gender	Age	Social class
1	6	Male	22–30	ABC1^a^
2	6	Female	22–30	C2DE
3	6	Male	31–47	C2DE
4	6	Female	48–60	ABC1
5	6	Male	48–60	C2DE
6	6	Female	60+	ABC1

*^a^This is a well-established demographic categorization used for market research purposes. It is based on occupation with ABC1 and C2DE taken to mean middle class and working class, respectively*.

Six focus group interviews were conducted in total with six participants in each. Based on suggestions by Guest et al. (39) that theoretical saturation can be reached between 12 and 15 in-depth observations and the coverage of socio-demographic factors (see Table 1) that can be achieved with such a number, this was deemed adequate *a priori*, and was subsequently confirmed upon analysis.

A semi-structured interview guide provided a framework for the focus groups’ discussions. (This is available on request from the authors.) Following a general discussion about of offal, product stimuli representing various combinations of ingredients (lung and heart), processing (mincing, freeze-drying, and extraction), and carrier product (mince beef, breakfast cereal^1^) were presented to each group (see Table 2). The ingredients were selected to represent ingredients with higher (heart) and lower (lung) levels of familiarity as edible products. The varying levels of processing were selected to present ingredients that are more (mince) and less (extracted) identifiable as originating from the associated raw materials. Furthermore, they represented different forms of transformation (physical and chemical).

**Table 2:** **Description of processing of beef by-products used for product concepts**.

(1) *Low level of processing*
Incorporation of ingredients into the carrier product through the process of mincing and mixing into the carrier product
(2) *Intermediate level of processing*
Incorporation of ingredients into the carrier products through a freeze-drying (lyophilization) process, where ingredients would be initially frozen, then dehydrated and crushed to produce a fine powder that would be mixed into the carrier product (similar to protein powders). (This process was used by Moreira-Araújo et al. (40) in developing a fortified food using beef lung.)
(3) *Highest level of processing*
Vitamin and mineral are extracted from offals and mixed into the proposed product concepts in the form of a concentrate. [Such a process could be used to produce functional foods containing bioactive peptides (12).]

All focus groups were recorded and transcribed verbatim. Thematic analysis using an inductive approach, following Braun and Clarke (41), was undertaken on the resulting data with the support of NVivo 10 (QSR International Ltd.). This involves identifying, coding, analyzing, and reporting themes within the data and interpreting these emerging themes in the context of research questions (41). In practice this mean familiarization with the data through reading and rereading transcripts; identifying items of interest, compiling, and designing the NVivo database; designing a coding framework and generating initial codes, grouping codes, and searching for themes, reviewing emerging themes; defining and naming higher order themes; and constructing and reporting these. Trustworthiness of the thematic analysis was ensured by engaging an independent researcher to code extracts and identify themes from the transcripts (42). Inter-coder reliability was high, as evidenced from a Cohen’s Kappa coefficient at 97%. Ethical approval was obtained from the UCC Social Research Committee prior to commencement of fieldwork.

## Results

The results are presented below according to the key themes that emerged during the analysis. Verbatim quotes from individuals within the focus groups are given where they serve to elaborate a point.

### Ideational Influences

Suggestions of eating offal lead to visceral (“*make my stomach turn*” Saoirse) and emotional responses among those with no previous experience of eating such organs. These affect based responses leveraged existing networks of meanings. The beating heart and breathing lung were associated with the living animal and thus responses were based on thoughts of consuming living organs. “*I feel like I would have a heart in your stomach* … *I actually would* … *and the beating* …” Saoirse (27). The idea of what the food represented was cause for rejection and, for some, it resulted in a very negative affective response:

“… you don’t really think about the bigger picture that it is an animal and that is the organ … and then you bring in the other organs that we are not used to it is like, that’s disgusting …” Emma (22)

The significance of early childhood experience on responses was very evident as illustrated in the case of Claire (28) who had eaten liver but not heart as a child. In the case of liver, she was unquestioning of its appropriateness as a food: “*I would try it again [liver] because I only had it when I was a child and I remember that I did like it*” while her response to eating heart was one of rejection “*I would be disgusted* ….” Furthermore, willingness to serve offal was also linked to ideational factors. Parents expected their children (due to lack of exposure to the offal) to respond negatively to being served offal “*I think my children would have a heart attack if I put that in front of them* …” Una (48) and as a result were somewhat unwilling to use these foods. Indeed, some suggested that to overcome expected rejection by their children, due to ideation around the product, they would disguise the offal within a dish, for example, adding spices and not revealing the ingredient.

In addition to disgust responses, questions on appropriateness were raised for offal that were generally considered inedible within Irish culture. Having no evidence or information on the value and benefits of eating the organ generated a negative perception as was evident in Emma’s (22) comment “… *everything in my brain says there are no benefits for me*.” Furthermore connections to the function and structure of the organ led to inferences on a potentially negative eating experience “*more rubbery because it is a muscle*” Paul (29).

A contamination response was also evident in the rejection of integrating fresh offal into fresh meat. This led to thoughts of nausea “… *yeah, I think it would be sickening* …” Claire (28). Thus incorporation of fresh offal into a widely used and liked food can lead to the rejection of that food. This contamination response was further illustrated through thoughts on family responses to covert exposure to the offal “*But if I told them after they eat it they probably wouldn’t eat mince again even there was nothing in it, so you would have to not tell them*.” Timothy (55).

However, early life experience with the offal resulted in discussions around taste and cooking approaches rather than ideational factors “*I’d thin slice it [heart] and cook it in the pan* …” Alan (50). In such cases there was an openness to new uses for fresh offal “*I would have no problem with that* … *and generally if I am going in I ask them to mince my beef* … *if I got a recipe to put heart through it I would* …” Patricia (60).

### Transformational Influences

Overcoming negative ideations around offal could occur through the use of processing. This “de-animalizes” the organ “*Like taking the nutrients out of the heart and actually not putting the heart meat into the mince* …” Sue (22). Higher levels of processing bring about the transformation of the offal from something impure, and possibly offensive, to something that is acceptable. Thus offering for sale vitamins extracted from the heart/lung was not as readily rejected on ideational grounds: “*If somebody gave me vitamin E powder from heart* … *don’t care that it came from heart*” Sinead (24). However, while “de-animalizing” the organ increases acceptance at one level, processing into ingredients also lead to negative evaluations with many associating processing with a reduction in naturalness and negative health outcomes “… *the more processed the food* … *it has a really bad name* … *in the last 10 years or so* … *everybody knows that the word processed is associated with something, it means it is not as good, like for me anyway, chemicals, but I know processed food is generally* … *you are always told to stay away from processed food* … *if anybody is giving anybody health advice* …” Paul (29). Given this association the carrier–ingredient combination created some conflicts in the minds of the consumer. In particular incorporating highly processed ingredient into a fresh meat (in the production process) raised questions on perceived necessity “*I think if people actually like heart it would be pointless* …” Timothy (55), and suspicion “*Yeah I don’t know* … *I just don’t like the idea of it full stop* … *they are trying to make more money out of each cow* …” Claire (28). The alignment between processing and carrier product was further illustrated in a openness to consuming offal extracts sold in capsule form. Niall (59) reasoned that extracts from offal “*in a capsule it is different you know* … *but pouring it into a* … *ugh* … *the thoughts of it being in a* …” while Claire (28) suggested that she “… *would buy heart vitamins* … *if they said all of these are great for your skin* … *in capsule form or* … *but I wouldn’t like the idea of it in food* ….” Use as a supplement generally appeared to result in a shift from thinking about the integration of a highly processed ingredient into a natural healthy food to the integration of a natural ingredient into a highly processed product. Thus what is judged as unhealthy and unnatural in one carrier product can be judged as health enhancing and beneficial in another. However, responses were not homogenous.

Past experience supported how sense was made of transformation processes and resultant ingredients. Some, for example, associated the inclusion of powdered heart in fresh meat with added preservatives and others made associations with existing ingredients used in the preparation of meals. While the latter lead to more positive evaluations the former resulted in the opposite. Creating ingredients from offal in similar forms to those traditionally used in the preparation of meat dishes increased acceptance among some older males “*see we would have grown up with Oxo cubes and a lot of that stuff you know* … *bisto* … *and it was all right then it was all right now* …” David (52). Linking powdered heart to crushed vitamin tablets by younger males made them more open to this being added to fresh meat “*I don’t think that there would be any problem if you went around and said here is mince with 100% more added vitamins, blah, blah, blah* … *once it doesn’t affect taste*” Tommy (25). Capsules were commonly associated with specific health benefits and extracts from food sources. Orla (63) speaks to this in reflecting back to her childhood and consideration of what her daughter should do now “… *when I was going to school my mother always gave us* … *and we were well fed* … *[plus a]* … *little halibut oil* … *it was yellow* … *and that was your vitamins* … *and I think it is going to go back to that because my daughter and my grandson were at the doctor there a while ago* … *and he wasn’t too well* … *and I said get him a tonic* … *and we were always well fed* … *but we also got our vitamins* ….” This evidence suggests that the past experiences and current thoughts on food influence overall evaluations through consideration of the organ, process, ingredient and carrier product, and combinations of same. Indeed familiarity with the form of the carrier was significant in overcoming ideational influences.

### Benefit Influences

Strong benefits are necessary to overcoming initial negative notions about the product. The strength and relevance of taste, health, and image benefits were central to acceptance. Emma (22) illustrated this in her shift from rejection of offal related products on ideation grounds to openness to trying when it was suggested that offal could improve hair, skin, nail, and/or muscle condition “*I would eat it yeah* … *I would give it a go anyway* … *like if it was disgusting* … *I would still give it a go* ….” However many were unwilling to trade these benefits if taste was comprised “*If it tasted nice* … *then I think I would try it again* … *and I’d prefer it but if it didn’t taste nice from the first one* … *even with all the benefits* … *I just think it would be a no go* ….” Emma (22).

Benefits were also central to acceptance for those who did not reject the products on ideational grounds. Simon (28) was not adverse to the idea of eating offal derived foods but would “… *need to put something beside it like the benefits of eating heart over something else* ….” Price, taste and performance benefits were relevant to these evaluations and most were unwilling to trade eating enjoyment for other benefits. If accepted at the ideational level and taste is acceptable then health benefits could command a price premium. However, if offal is seen as an inferior product, a waste product (being used to bulk up the meat), then suggestions of a premium were rejected irrespective of the other benefits it offered “*I’d expect it to be cheaper* … *because I would consider the heart to be kind of like offal or that it would be cheaper than regular mince so* …. *I would have thought it would be a cheaper pack* …” Johnny (26).

Credibility of the benefit claim and safety of the product was key “*Yeah but if it is going to have heart in it* … *it’s not going to be like* … *it would have to be [from] someone that is completely trustworthy and* ….” Aoife (26). Furthermore, external verification of claims from a trusted agency enhanced acceptance “[company CEO name] *is not going to sell this idea to me* … *because I know that he is only doing this for money* … *but if Bord Bia may sell it to me because I trust them as a government agency to do what is right by the consumer* …” Kevin (41).

Table 3 summarizes the factors influencing acceptance and rejection of the three offal-derived product concepts.

**Table 3:** **Influences on acceptance and rejection of offal derived foods**.

	Meat plus offal	Mince plus powder offal	Concentrated extract
Reasons for rejection	Ideational Emotional and visceral responses Appropriateness	Non-alignment between level of processing and product	Necessity
	Negative taste experience	Negative health perceptions due to levels of processing	
	Industry motivations questioned	Necessity	
Reasons for accepting	Past experience	Get health benefit	Control
	Liking taste	Could be like a seasoning	Transparency
			Clear benefit
			Natural ingredient
Prerequisite to acceptance	Trust in oversight that the products are safe		

## Discussion

The influence of how a food was produced and processed on consumer product evaluations is well established (43–46). Our findings indicate that the physical state of the offal as a result of different processing technologies, and the extent to which it could be identified as the source of the ingredient influences acceptance. This is in keeping with Hartmann et al. (32) who found that acceptance of insect ingredients is influenced by the visibility of the insect ingredients, and Martins and Pliner (22) who found that distancing an ingredient of animal origin from its original state so as to render it into an unrecognizable state (and to “de-animalize it”) influenced acceptance. In this way, the research speaks to the use of physical state as a heuristic to evaluate food. Our research also found a link between physical state and perceived healthiness. In contrast to Szocs and Lefebvre (30), who argue that the blender effect is “linear” when they investigated the effect of physical state on perceived healthiness and calorie content (more processed means less healthy and higher calories), we concur with Gmuer et al. (29) in arguing that the relationship between physical state and acceptance is much more complex. We found a few examples of where higher levels of processing did not necessarily result in higher greater levels of acceptance, e.g., concentrated extracts would be accepted if they offered a benefit. Association of the more processed extracts with supplements and as a result with greater acceptance can be rationalized by Rozin et al. (25) finding that preference for naturalness is substantial and stronger in foods than for medicines. They find a natural preference exists for foods but not for medicines with a suggestion that products such as vitamins, that are transitional between food and medicine, fall between foods and medicines in natural preferences. In relation to opportunities for offal, the finding that a supplement containing a food extract is seen as more natural than the other alternatives points to some product development opportunities for those interested in supplements as part of the solution to the health needs.

Responses to the proposed use of offal in food, and as part of a healthy eating solution, appear to vary based on demographic characteristics. This draws attention to the potential impact of life experiences on interpretations and evaluations of products and processes. Older participants were more open to the idea of consuming offal in forms close to its original state due to their early life experiences with these and similar products. Some younger males displayed a degree of “machoism” in their responses with suggestions that while they would eat this, other family members might be offended. This might, in part, be explained by these younger men acting out the role associated with hegemonic masculinity (47). Indeed, it was women, and particularly younger women, that displayed the most negative responses. This could speak to both the influence of gender identity and lack of familiarity on evaluations. This observed gender difference is in keeping with the work of Verbeke (48) who noted that females were more likely to be negative with regard to novel foods. Clearly both food and non-food life experience can influence responses to novel foods and it is through familiarity with these that evaluations move away from ideational barriers and toward product characteristics and benefits. This suggests that time is an important factor in determining consumer acceptance. The introduction to growth phase of the product lifecycle could be protracted as these products are demanding a change in consumer perception of what constitutes a food. Acceptance may increase with ongoing exposure to the use of offal based products by others.

Martins and Pliner (23) highlight that the motivational dimensions underlying acceptance/rejection are bipolar. This suggests that focusing on the positive pole and inducing positive motives may encourage acceptance and thus it is important to identify core motives. This research has identified rejection motivations relating to distaste, danger and the ideational factors of disgust and inappropriateness across all product concepts. Thus the challenge is to change perceptions of, for example, distaste to taste, harmful to healthy, aversive texture to acceptable, while addressing neophobic responses. Cultural context also needs to be considered as Ruby and Heine (19) found in relation to investigating factors influencing people’s decision to eat different type of animals.

Following this view of inducing positive motives, evidence of different levels of acceptance across consumer segments, and low levels of knowledge/familiarity, these authors suggest that a research commercialization strategy for adding value to offal should focus on specific market segments initially. Following House (49), we also suggest that generating general acceptability must build on a degree of established consumption which should “not emphasise *reducing or changing negative attitudes* in the general population, but *increasing the positive and distinctive attributes* of [such foods], such as their taste,” i.e., messaging should focus on positive links. This along with the earlier observation about the long lead time suggests that the first products to be introduced should be designed around a benefit that is important to those more open to offal based product and that the messaging should include the notion that this solution is also morally relevant and desirable. Such an approach is designed to result in a relatively small but established number of repeat consumers upon which future more widespread consumption would be build. In the context of offal, early adopters could be males interested in physical performance and body image who have experience with using supplements or older male who have fond recollections of offal. The time and effort however required by interested stakeholders to effect this change will be considerable.

Such an approach also needs to focus on the factors which are likely to generate repeat purchase as opposed to one-off trial. This requires having a greater understanding of the social, practical, and contextual factors that influence *repeat* purchase as opposed to one-off trial in addition to the individual factors that affect acceptance of novel foods. This research highlights the importance of such factors in relation to offal and identified degree of fit with existing culinary practices and knowledge, and degree of fit with established dietary practices, including accommodating other household members’ preferences as influential. Findings regarding the influence of degree of fit with existing culinary practices and knowledge were also found to be an important determinant of acceptance by Looy et al. (50) in relation to insects. The fact that products were presented in this research as ingredients rather than as finished foods was important. Ingredients give consumers flexibility in terms of how they can be integrated into their routines with consumers in this research identifying that some ingredients could be integrated into their culinary routines through use as seasoning for example. Following acceptance by early adopters, greater acceptance can built on this through exposure on the basis that exposure to such products in stores and restaurants indicates that other people are consuming these products and that such products are safe and socially acceptable (19). Experts, celebrity chefs, and friends have an important role to play in reducing consumer concerns by providing an example of “correct” behavior that people can copy, i.e., providing “social proof.”

Based on these findings, the following are proposed as pathways toward commercialization/increased use of beef by-products:

1. Develop products that can be used as ingredients rather than as finished products so that they can be integrated into existing routines and thus encourage greater or more regular uptake.
2. Incorporate more familiar product and ensure compatibility between ingredient and carrier if the ingredient will be visible.
3. Consider the perceived naturalness of the ingredient and the associated processing technology and ensure a fit between consumer required level of naturalness and ingredient application.
4. Focus on early adopters, address supply, and availability issues as well as obvious ones such as price, taste, and benefit.
5. Utilize a processing technology that is associated with benefits.
6. Increase awareness of range of by-products that are edible/have edible/food grade constituents.
7. Focus social proof around celebrities and chefs.

This research highlights the significant influence of ideation on acceptance/rejection of products that incorporate ingredients derived from offal. It also highlights the difference in acceptance among different consumer segments, with segmentation based on demographics having salience. In particular, it highlights the significant role that industry has to play in carefully designing products, developing targeted marketing strategies, and ensuring such products are widely available if value added products derived from offal are to become an accepted and integrated part of people’s diets, since changes in values are often supply driven (51).

## Author Contributions

MH and MM were awarded the research funding for the study. JO worked under their supervision to design the study and develop the methodology. MM performed the analysis, and MH led on writing the manuscript. JO collected the data.

## Conflict of Interest Statement

The authors declare that the research was conducted in the absence of any commercial or financial relationships that could be construed as a potential conflict of interest.

## Footnotes

^1^The results relating to the cereal product are not presented in this paper.
